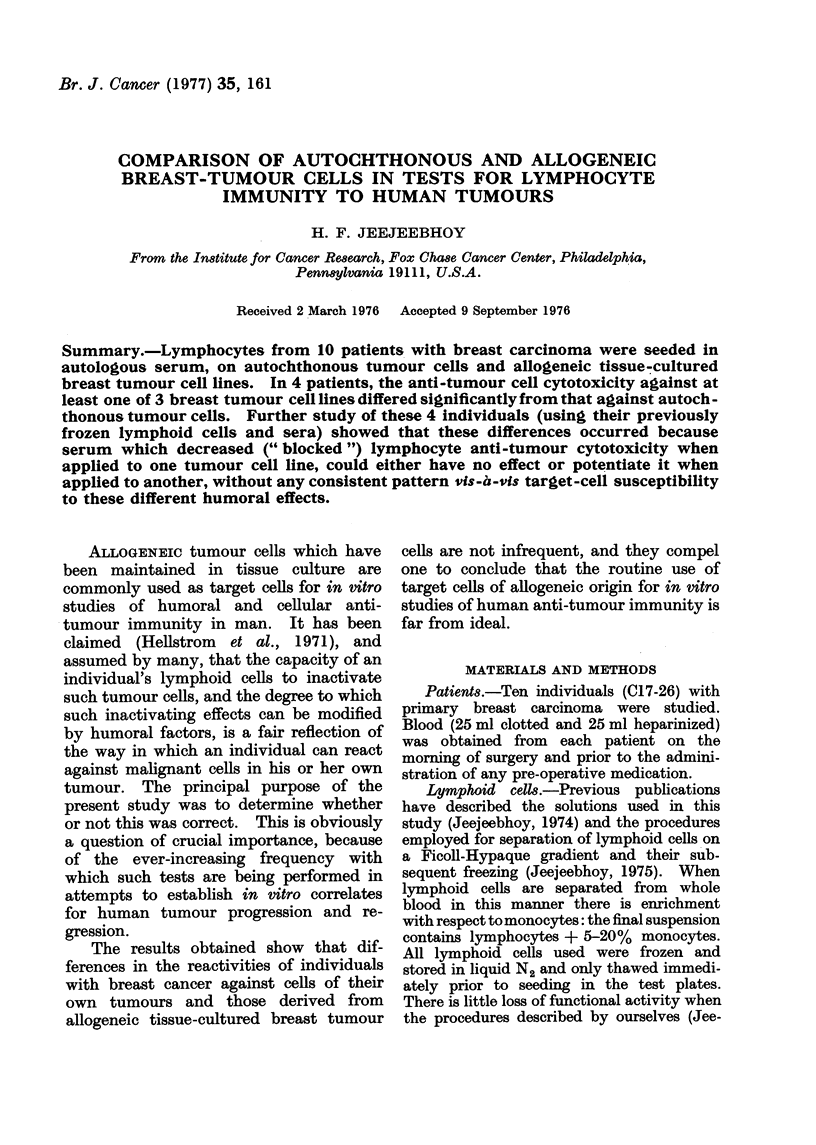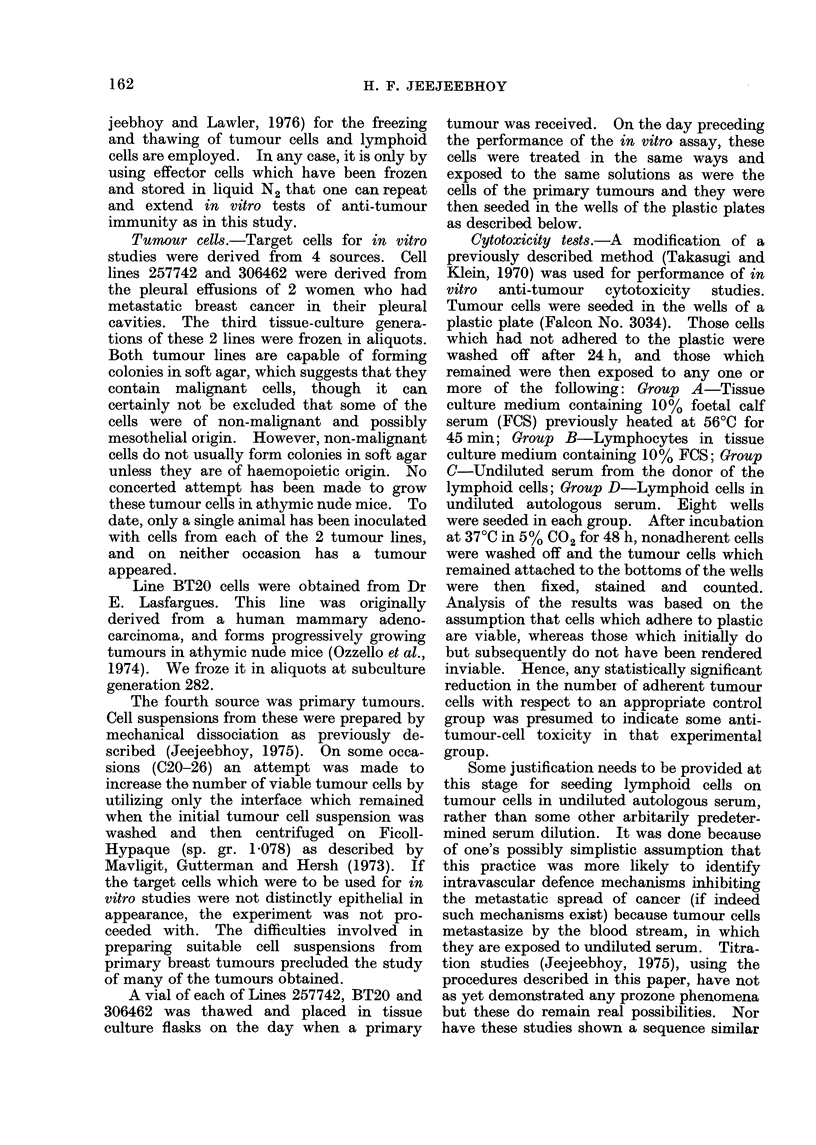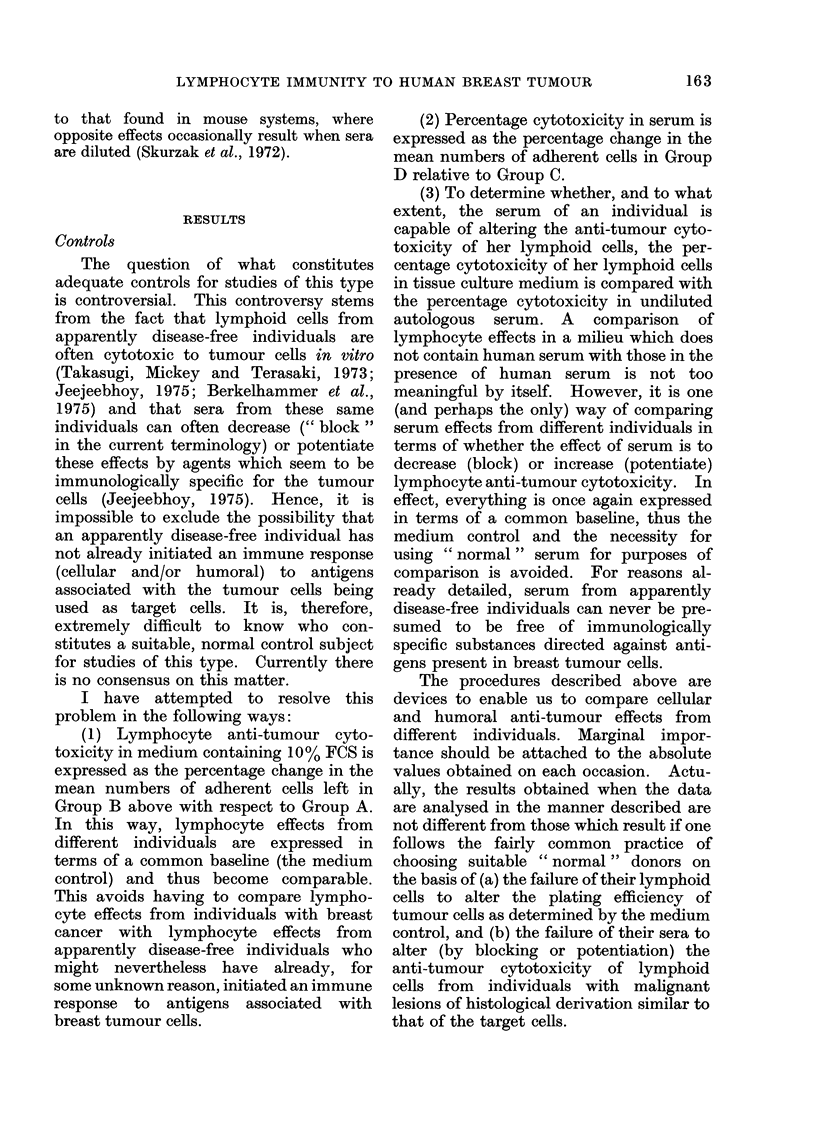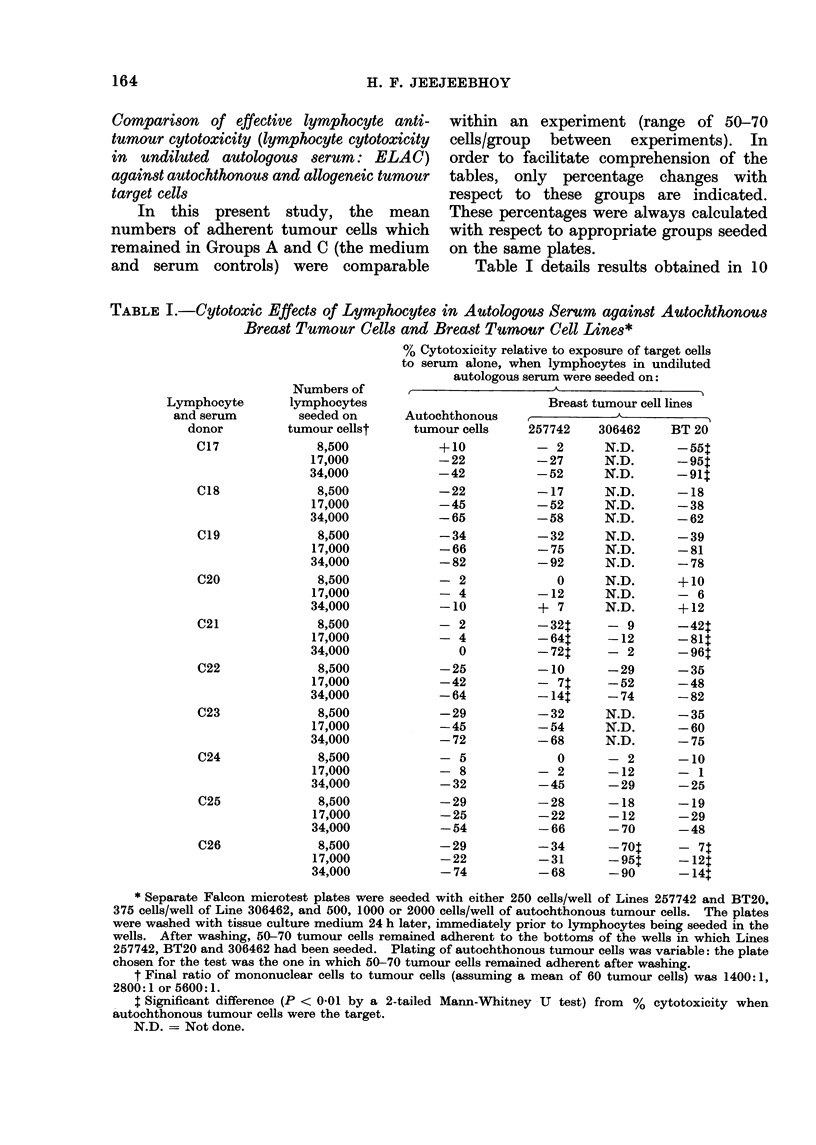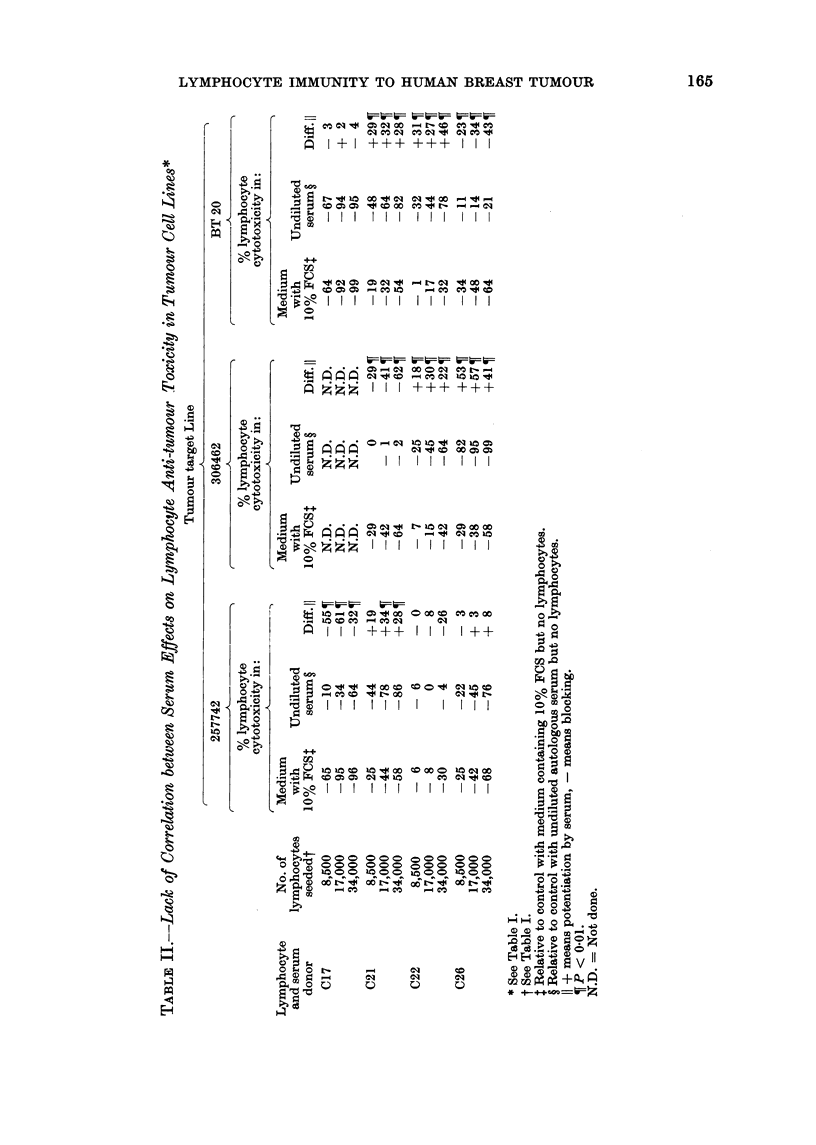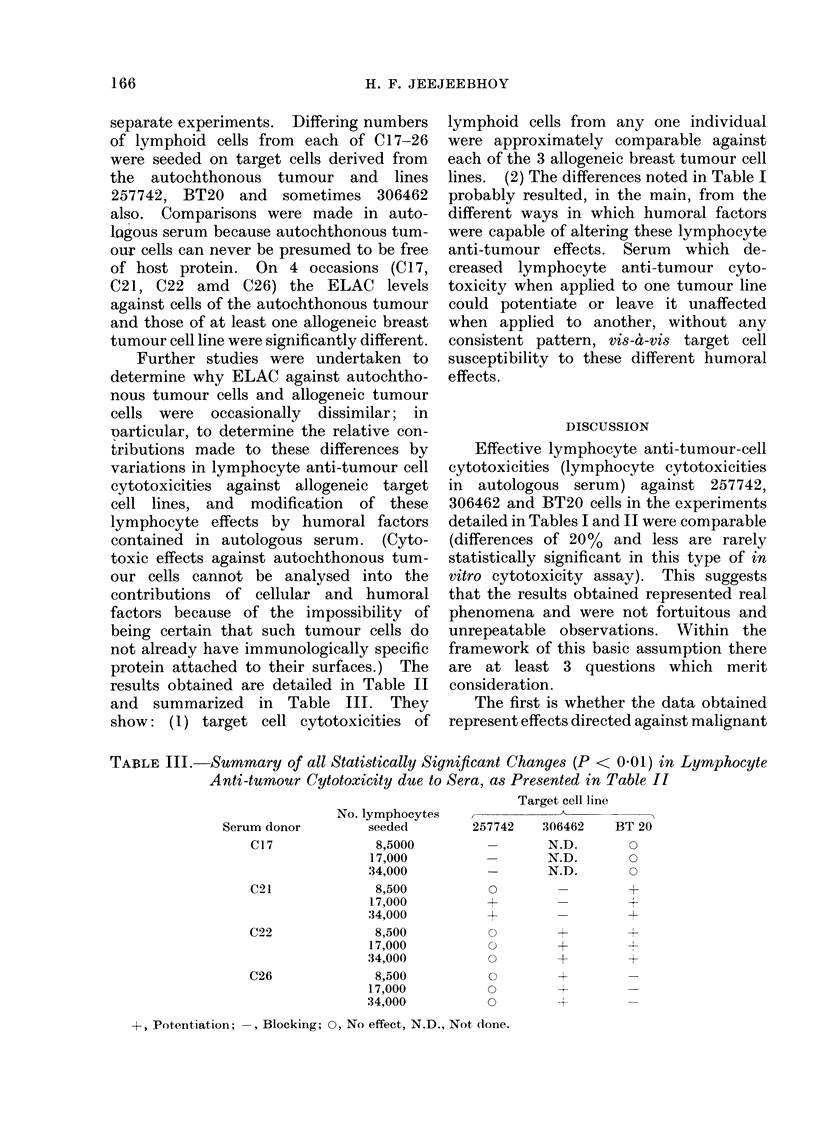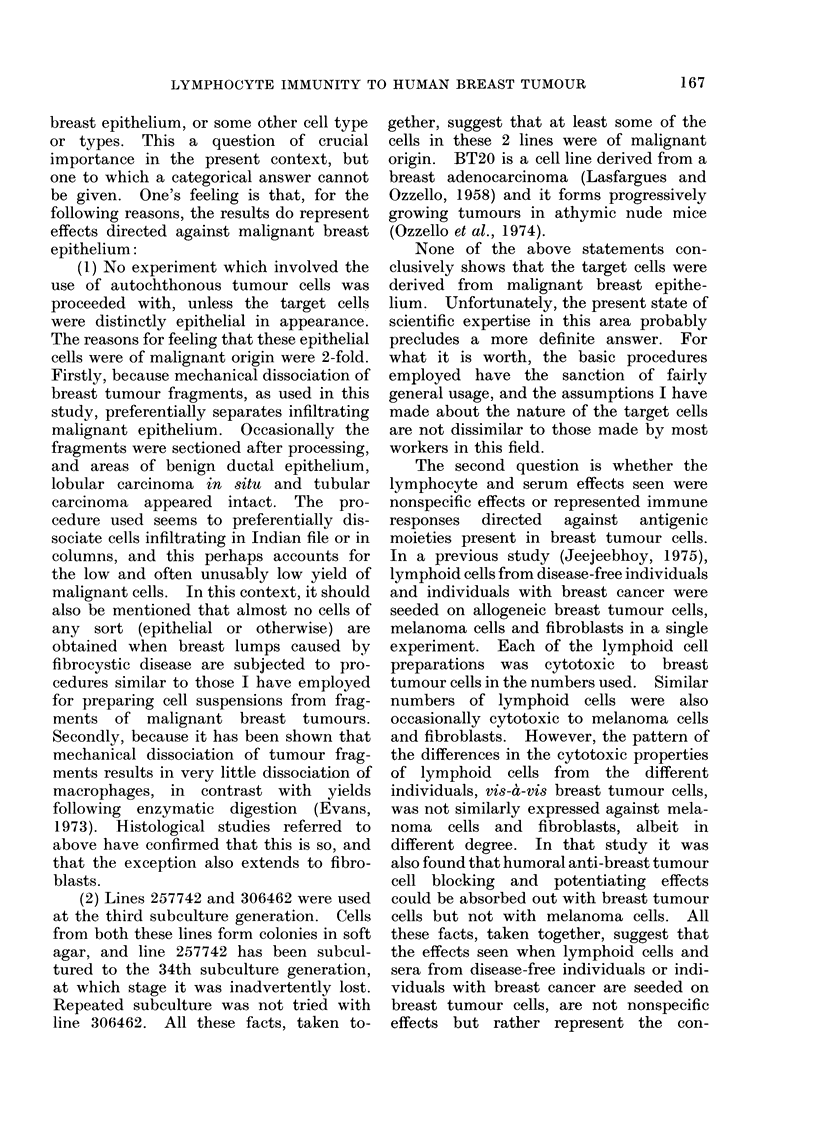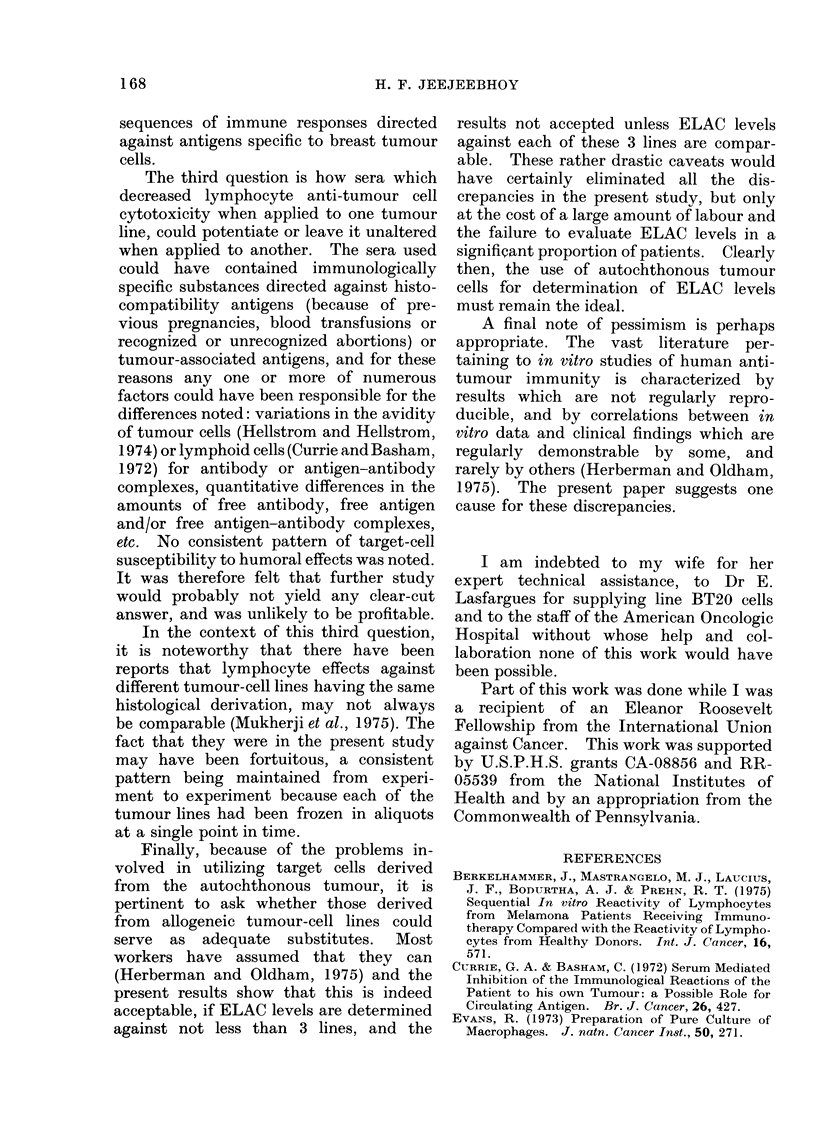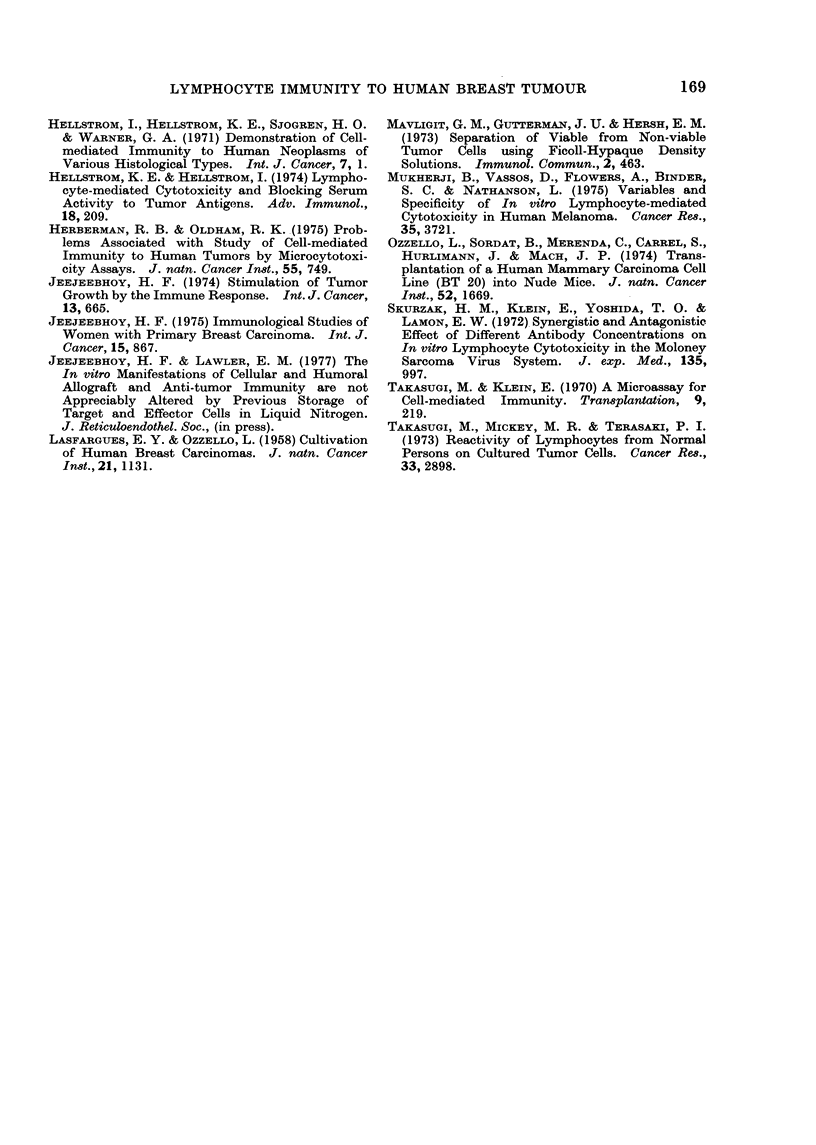# Comparison of autochthonous and allogeneic breast-tumour cells in tests for lymphocyte immunity to human tumours.

**DOI:** 10.1038/bjc.1977.23

**Published:** 1977-02

**Authors:** H. F. Jeejeebhoy

## Abstract

Lymphocytes from 10 patients with breast carcinoma were seeded in autologous serum, on autochthonous tumour cells and allogeneic tissue-cultured breast tumour cell lines. In 4 patients, the anti-tumour cell cytotoxicity against at least one of 3 breast tumour cell lines differed significantly from that against autochthonous tumour cells. Further study of these 4 individuals (using their previously frozen lymphoid cells and sera) showed that these differences occurred because serum which decreased ("blocked") lymphocyte anti-tumour cytotoxicity when applied to one tumour cell line, could either have no effect or potentiate it when applied to another, without any consistent pattern vis-à-vis target-cell susceptibility to these different humoral effects.


*Br. J. Cancer* (1977) **35**, 161

### COMPARISON OF AUTOCHTHONOUS AND ALLOGENEIC BREAST-TUMOUR CELLS IN TESTS FOR LYMPHOCYTE IMMUNITY TO HUMAN TUMOURS

H. F. JEEJEEBHOY

*From the Institute for Cancer Research, Fox Chase Cancer Center, Philadelphia, Pennsylvania* 19111, *U.S.A.*

Received 2 March 1976 Accepted 9 September 1976

**Summary.—Lymphocytes from 10 patients with breast carcinoma were seeded in autologous serum, on autochthonous tumour cells and allogeneic tissue-cultured breast tumour cell lines. In 4 patients, the anti-tumour cell cytotoxicity against at least one of 3 breast tumour cell lines differed significantly from that against autochthonous tumour cells. Further study of these 4 individuals (using their previously frozen lymphoid cells and sera) showed that these differences occurred because serum which decreased (" blocked ") lymphocyte anti-tumour cytotoxicity when applied to one tumour cell line, could either have no effect or potentiate it when applied to another, without any consistent pattern *vis-à-vis* target-cell susceptibility to these different humoral effects.**

ALLOGENEIC tumour cells which have been maintained in tissue culture are commonly used as target cells for *in vitro* studies of humoral and cellular anti-tumour immunity in man. It has been claimed (Hellstrom *et al.*, 1971), and assumed by many, that the capacity of an individual's lymphoid cells to inactivate such tumour cells, and the degree to which such inactivating effects can be modified by humoral factors, is a fair reflection of the way in which an individual can react against malignant cells in his or her own tumour. The principal purpose of the present study was to determine whether or not this was correct. This is obviously a question of crucial importance, because of the ever-increasing frequency with which such tests are being performed in attempts to establish *in vitro* correlates for human tumour progression and regression.

The results obtained show that differences in the reactivities of individuals with breast cancer against cells of their own tumours and those derived from allogeneic tissue-cultured breast tumour cells are not infrequent, and they compel one to conclude that the routine use of target cells of allogeneic origin for *in vitro* studies of human anti-tumour immunity is far from ideal.

### MATERIALS AND METHODS

*Patients.*—Ten individuals (C17-26) with primary breast carcinoma were studied. Blood (25 ml clotted and 25 ml heparinized) was obtained from each patient on the morning of surgery and prior to the administration of any pre-operative medication.

*Lymphoid cells.*—Previous publications have described the solutions used in this study (Jeejeebhoy, 1974) and the procedures employed for separation of lymphoid cells on a Ficoll-Hypaque gradient and their subsequent freezing (Jeejeebhoy, 1975). When lymphoid cells are separated from whole blood in this manner there is enrichment with respect to monocytes: the final suspension contains lymphocytes + 5–20% monocytes. All lymphoid cells used were frozen and stored in liquid $N_2$ and only thawed immediately prior to seeding in the test plates. There is little loss of functional activity when the procedures described by ourselves (Jeejeebhoy and Lawler, 1976) for the freezing and thawing of tumour cells and lymphoid cells are employed. In any case, it is only by using effector cells which have been frozen and stored in liquid $N_2$ that one can repeat and extend *in vitro* tests of anti-tumour immunity as in this study.

*Tumour cells.*—Target cells for *in vitro* studies were derived from 4 sources. Cell lines 257742 and 306462 were derived from the pleural effusions of 2 women who had metastatic breast cancer in their pleural cavities. The third tissue-culture generations of these 2 lines were frozen in aliquots. Both tumour lines are capable of forming colonies in soft agar, which suggests that they contain malignant cells, though it can certainly not be excluded that some of the cells were of non-malignant and possibly mesothelial origin. However, non-malignant cells do not usually form colonies in soft agar unless they are of haemopoietic origin. No concerted attempt has been made to grow these tumour cells in athymic nude mice. To date, only a single animal has been inoculated with cells from each of the 2 tumour lines, and on neither occasion has a tumour appeared.

Line BT20 cells were obtained from Dr E. Lasfargues. This line was originally derived from a human mammary adenocarcinoma, and forms progressively growing tumours in athymic nude mice (Ozzello *et al.*, 1974). We froze it in aliquots at subculture generation 282.

The fourth source was primary tumours. Cell suspensions from these were prepared by mechanical dissociation as previously described (Jeejeebhoy, 1975). On some occasions (C20–26) an attempt was made to increase the number of viable tumour cells by utilizing only the interface which remained when the initial tumour cell suspension was washed and then centrifuged on Ficoll-Hypaque (sp. gr. 1·078) as described by Mavligit, Gutterman and Hersh (1973). If the target cells which were to be used for *in vitro* studies were not distinctly epithelial in appearance, the experiment was not proceeded with. The difficulties involved in preparing suitable cell suspensions from primary breast tumours precluded the study of many of the tumours obtained.

A vial of each of Lines 257742, BT20 and 306462 was thawed and placed in tissue culture flasks on the day when a primary tumour was received. On the day preceding the performance of the *in vitro* assay, these cells were treated in the same ways and exposed to the same solutions as were the cells of the primary tumours and they were then seeded in the wells of the plastic plates as described below.

*Cytotoxicity tests.*—A modification of a previously described method (Takasugi and Klein, 1970) was used for performance of *in vitro* anti-tumour cytotoxicity studies. Tumour cells were seeded in the wells of a plastic plate (Falcon No. 3034). Those cells which had not adhered to the plastic were washed off after 24 h, and those which remained were then exposed to any one or more of the following: *Group A*—Tissue culture medium containing 10% foetal calf serum (FCS) previously heated at 56°C for 45 min; *Group B*—Lymphocytes in tissue culture medium containing 10% FCS; *Group C*—Undiluted serum from the donor of the lymphoid cells; *Group D*—Lymphoid cells in undiluted autologous serum. Eight wells were seeded in each group. After incubation at 37°C in 5% $CO_2$ for 48 h, nonadherent cells were washed off and the tumour cells which remained attached to the bottoms of the wells were then fixed, stained and counted. Analysis of the results was based on the assumption that cells which adhere to plastic are viable, whereas those which initially do but subsequently do not have been rendered inviable. Hence, any statistically significant reduction in the number of adherent tumour cells with respect to an appropriate control group was presumed to indicate some anti-tumour-cell toxicity in that experimental group.

Some justification needs to be provided at this stage for seeding lymphoid cells on tumour cells in undiluted autologous serum, rather than some other arbitarily predetermined serum dilution. It was done because of one's possibly simplistic assumption that this practice was more likely to identify intravascular defence mechanisms inhibiting the metastatic spread of cancer (if indeed such mechanisms exist) because tumour cells metastasize by the blood stream, in which they are exposed to undiluted serum. Titration studies (Jeejeebhoy, 1975), using the procedures described in this paper, have not as yet demonstrated any prozone phenomena but these do remain real possibilities. Nor have these studies shown a sequence similar to that found in mouse systems, where opposite effects occasionally result when sera are diluted (Skurzak *et al.*, 1972).

### RESULTS

### *Controls*

The question of what constitutes adequate controls for studies of this type is controversial. This controversy stems from the fact that lymphoid cells from apparently disease-free individuals are often cytotoxic to tumour cells *in vitro* (Takasugi, Mickey and Terasaki, 1973; Jeejeebhoy, 1975; Berkelhammer *et al.*, 1975) and that sera from these same individuals can often decrease (" block " in the current terminology) or potentiate these effects by agents which seem to be immunologically specific for the tumour cells (Jeejeebhoy, 1975). Hence, it is impossible to exclude the possibility that an apparently disease-free individual has not already initiated an immune response (cellular and/or humoral) to antigens associated with the tumour cells being used as target cells. It is, therefore, extremely difficult to know who constitutes a suitable, normal control subject for studies of this type. Currently there is no consensus on this matter.

I have attempted to resolve this problem in the following ways:

(1) Lymphocyte anti-tumour cytotoxicity in medium containing 10% FCS is expressed as the percentage change in the mean numbers of adherent cells left in Group B above with respect to Group A. In this way, lymphocyte effects from different individuals are expressed in terms of a common baseline (the medium control) and thus become comparable. This avoids having to compare lymphocyte effects from individuals with breast cancer with lymphocyte effects from apparently disease-free individuals who might nevertheless have already, for some unknown reason, initiated an immune response to antigens associated with breast tumour cells.

(2) Percentage cytotoxicity in serum is expressed as the percentage change in the mean numbers of adherent cells in Group D relative to Group C.

(3) To determine whether, and to what extent, the serum of an individual is capable of altering the anti-tumour cytotoxicity of her lymphoid cells, the percentage cytotoxicity of her lymphoid cells in tissue culture medium is compared with the percentage cytotoxicity in undiluted autologous serum. A comparison of lymphocyte effects in a milieu which does not contain human serum with those in the presence of human serum is not too meaningful by itself. However, it is one (and perhaps the only) way of comparing serum effects from different individuals in terms of whether the effect of serum is to decrease (block) or increase (potentiate) lymphocyte anti-tumour cytotoxicity. In effect, everything is once again expressed in terms of a common baseline, thus the medium control and the necessity for using " normal " serum for purposes of comparison is avoided. For reasons already detailed, serum from apparently disease-free individuals can never be presumed to be free of immunologically specific substances directed against antigens present in breast tumour cells.

The procedures described above are devices to enable us to compare cellular and humoral anti-tumour effects from different individuals. Marginal importance should be attached to the absolute values obtained on each occasion. Actually, the results obtained when the data are analysed in the manner described are not different from those which result if one follows the fairly common practice of choosing suitable " normal " donors on the basis of (a) the failure of their lymphoid cells to alter the plating efficiency of tumour cells as determined by the medium control, and (b) the failure of their sera to alter (by blocking or potentiation) the anti-tumour cytotoxicity of lymphoid cells from individuals with malignant lesions of histological derivation similar to that of the target cells.

*Comparison of effective lymphocyte anti-tumour cytotoxicity (lymphocyte cytotoxicity in undiluted autologous serum: ELAC) against autochthonous and allogeneic tumour target cells*

In this present study, the mean numbers of adherent tumour cells which remained in Groups A and C (the medium and serum controls) were comparable within an experiment (range of 50–70 cells/group between experiments). In order to facilitate comprehension of the tables, only percentage changes with respect to these groups are indicated. These percentages were always calculated with respect to appropriate groups seeded on the same plates.

Table I details results obtained in 10

TABLE I.—*Cytotoxic Effects of Lymphocytes in Autologous Serum against Autochthonous Breast Tumour Cells and Breast Tumour Cell Lines**

| Lymphocyte and serum donor | Numbers of lymphocytes seeded on tumour cells† | % Cytotoxicity relative to exposure of target cells to serum alone, when lymphocytes in undiluted autologous serum were seeded on: Autochthonous tumour cells | Breast tumour cell lines: 257742 | 306462 | BT 20 |
|---|---|---|---|---|---|
| C17 | 8,500 | +10 | − 2 | N.D. | −55‡ |
| | 17,000 | −22 | −27 | N.D. | −95‡ |
| | 34,000 | −42 | −52 | N.D. | −91‡ |
| C18 | 8,500 | −22 | −17 | N.D. | −18 |
| | 17,000 | −45 | −52 | N.D. | −38 |
| | 34,000 | −65 | −58 | N.D. | −62 |
| C19 | 8,500 | −34 | −32 | N.D. | −39 |
| | 17,000 | −66 | −75 | N.D. | −81 |
| | 34,000 | −82 | −92 | N.D. | −78 |
| C20 | 8,500 | − 2 | 0 | N.D. | +10 |
| | 17,000 | − 4 | −12 | N.D. | − 6 |
| | 34,000 | −10 | + 7 | N.D. | +12 |
| C21 | 8,500 | − 2 | −32‡ | − 9 | −42‡ |
| | 17,000 | − 4 | −64‡ | −12 | −81‡ |
| | 34,000 | 0 | −72‡ | − 2 | −96‡ |
| C22 | 8,500 | −25 | −10 | −29 | −35 |
| | 17,000 | −42 | − 7‡ | −52 | −48 |
| | 34,000 | −64 | −14‡ | −74 | −82 |
| C23 | 8,500 | −29 | −32 | N.D. | −35 |
| | 17,000 | −45 | −54 | N.D. | −60 |
| | 34,000 | −72 | −68 | N.D. | −75 |
| C24 | 8,500 | − 5 | 0 | − 2 | −10 |
| | 17,000 | − 8 | − 2 | −12 | − 1 |
| | 34,000 | −32 | −45 | −29 | −25 |
| C25 | 8,500 | −29 | −28 | −18 | −19 |
| | 17,000 | −25 | −22 | −12 | −29 |
| | 34,000 | −54 | −66 | −70 | −48 |
| C26 | 8,500 | −29 | −34 | −70‡ | − 7‡ |
| | 17,000 | −22 | −31 | −95‡ | −12‡ |
| | 34,000 | −74 | −68 | −90 | −14‡ |

* Separate Falcon microtest plates were seeded with either 250 cells/well of Lines 257742 and BT20. 375 cells/well of Line 306462, and 500, 1000 or 2000 cells/well of autochthonous tumour cells. The plates were washed with tissue culture medium 24 h later, immediately prior to lymphocytes being seeded in the wells. After washing, 50–70 tumour cells remained adherent to the bottoms of the wells in which Lines 257742, BT20 and 306462 had been seeded. Plating of autochthonous tumour cells was variable: the plate chosen for the test was the one in which 50–70 tumour cells remained adherent after washing.

† Final ratio of mononuclear cells to tumour cells (assuming a mean of 60 tumour cells) was 1400:1, 2800:1 or 5600:1.

‡ Significant difference ($P < 0{\cdot}01$ by a 2-tailed Mann-Whitney U test) from % cytotoxicity when autochthonous tumour cells were the target.

N.D. = Not done.

TABLE II.—*Lack of Correlation between Serum Effects on Lymphocyte Anti-tumour Toxicity in Tumour Cell Lines**

| | | Tumour target Line | | | | | | | | |
|---|---|---|---|---|---|---|---|---|---|---|
| | | 257742 | | | 306462 | | | BT 20 | | |
| | | % lymphocyte cytotoxicity in: | | | % lymphocyte cytotoxicity in: | | | % lymphocyte cytotoxicity in: | | |
| Lymphocyte and serum donor | No. of lymphocytes seeded† | Medium with 10% FCS‡ | Undiluted serum§ | Diff.‖ | Medium with 10% FCS‡ | Undiluted serum§ | Diff.‖ | Medium with 10% FCS‡ | Undiluted serum§ | Diff.‖ |
| C17 | 8,500 | −65 | −10 | −55¶ | N.D. | N.D. | N.D. | −64 | −67 | − 3 |
| | 17,000 | −95 | −34 | −61¶ | N.D. | N.D. | N.D. | −92 | −94 | + 2 |
| | 34,000 | −96 | −64 | −32¶ | N.D. | N.D. | N.D. | −99 | −95 | − 4 |
| C21 | 8,500 | −25 | −44 | +19 | −29 | 0 | −29¶ | −19 | −48 | +29¶ |
| | 17,000 | −44 | −78 | +34¶ | −42 | − 1 | −41¶ | −32 | −64 | +32¶ |
| | 34,000 | −58 | −86 | +28¶ | −64 | − 2 | −62¶ | −54 | −82 | +28¶ |
| C22 | 8,500 | − 6 | − 6 | − 0 | − 7 | −25 | +18¶ | − 1 | −32 | +31¶ |
| | 17,000 | − 8 | 0 | − 8 | −15 | −45 | +30¶ | −17 | −44 | +27¶ |
| | 34,000 | −30 | − 4 | −26 | −42 | −64 | +22¶ | −32 | −78 | +46¶ |
| C26 | 8,500 | −25 | −22 | − 3 | −29 | −82 | +53¶ | −34 | −11 | −23¶ |
| | 17,000 | −42 | −45 | + 3 | −38 | −95 | +57¶ | −48 | −14 | −34¶ |
| | 34,000 | −68 | −76 | + 8 | −58 | −99 | +41¶ | −64 | −21 | −43¶ |

* See Table I.

† See Table I.

‡ Relative to control with medium containing 10% FCS but no lymphocytes.

§ Relative to control with undiluted autologous serum but no lymphocytes.

‖ + means potentiation by serum, — means blocking.

¶ $P < 0{\cdot}01$.

N.D. = Not done.

separate experiments. Differing numbers of lymphoid cells from each of C17–26 were seeded on target cells derived from the autochthonous tumour and lines 257742, BT20 and sometimes 306462 also. Comparisons were made in autologous serum because autochthonous tumour cells can never be presumed to be free of host protein. On 4 occasions (C17, C21, C22 amd C26) the ELAC levels against cells of the autochthonous tumour and those of at least one allogeneic breast tumour cell line were significantly different.

Further studies were undertaken to determine why ELAC against autochthonous tumour cells and allogeneic tumour cells were occasionally dissimilar; in particular, to determine the relative contributions made to these differences by variations in lymphocyte anti-tumour cell cytotoxicities against allogeneic target cell lines, and modification of these lymphocyte effects by humoral factors contained in autologous serum. (Cytotoxic effects against autochthonous tumour cells cannot be analysed into the contributions of cellular and humoral factors because of the impossibility of being certain that such tumour cells do not already have immunologically specific protein attached to their surfaces.) The results obtained are detailed in Table II and summarized in Table III. They show: (1) target cell cytotoxicities of lymphoid cells from any one individual were approximately comparable against each of the 3 allogeneic breast tumour cell lines. (2) The differences noted in Table I probably resulted, in the main, from the different ways in which humoral factors were capable of altering these lymphocyte anti-tumour effects. Serum which decreased lymphocyte anti-tumour cytotoxicity when applied to one tumour line could potentiate or leave it unaffected when applied to another, without any consistent pattern, *vis-à-vis* target cell susceptibility to these different humoral effects.

### DISCUSSION

Effective lymphocyte anti-tumour-cell cytotoxicities (lymphocyte cytotoxicities in autologous serum) against 257742, 306462 and BT20 cells in the experiments detailed in Tables I and II were comparable (differences of 20% and less are rarely statistically significant in this type of *in vitro* cytotoxicity assay). This suggests that the results obtained represented real phenomena and were not fortuitous and unrepeatable observations. Within the framework of this basic assumption there are at least 3 questions which merit consideration.

The first is whether the data obtained represent effects directed against malignant

TABLE III.—*Summary of all Statistically Significant Changes ($P < 0{\cdot}01$) in Lymphocyte Anti-tumour Cytotoxicity due to Sera, as Presented in Table II*

| Serum donor | No. lymphocytes seeded | Target cell line 257742 | 306462 | BT 20 |
|---|---|---|---|---|
| C17 | 8,5000 | — | N.D. | O |
| | 17,000 | — | N.D. | O |
| | 34,000 | — | N.D. | O |
| C21 | 8,500 | O | — | + |
| | 17,000 | + | — | + |
| | 34,000 | + | — | + |
| C22 | 8,500 | O | + | + |
| | 17,000 | O | + | + |
| | 34,000 | O | + | + |
| C26 | 8,500 | O | + | — |
| | 17,000 | O | + | — |
| | 34,000 | O | + | — |

+, Potentiation; —, Blocking; O, No effect, N.D., Not done.

breast epithelium, or some other cell type or types. This a question of crucial importance in the present context, but one to which a categorical answer cannot be given. One's feeling is that, for the following reasons, the results do represent effects directed against malignant breast epithelium:

(1) No experiment which involved the use of autochthonous tumour cells was proceeded with, unless the target cells were distinctly epithelial in appearance. The reasons for feeling that these epithelial cells were of malignant origin were 2-fold. Firstly, because mechanical dissociation of breast tumour fragments, as used in this study, preferentially separates infiltrating malignant epithelium. Occasionally the fragments were sectioned after processing, and areas of benign ductal epithelium, lobular carcinoma *in situ* and tubular carcinoma appeared intact. The procedure used seems to preferentially dissociate cells infiltrating in Indian file or in columns, and this perhaps accounts for the low and often unusably low yield of malignant cells. In this context, it should also be mentioned that almost no cells of any sort (epithelial or otherwise) are obtained when breast lumps caused by fibrocystic disease are subjected to procedures similar to those I have employed for preparing cell suspensions from fragments of malignant breast tumours. Secondly, because it has been shown that mechanical dissociation of tumour fragments results in very little dissociation of macrophages, in contrast with yields following enzymatic digestion (Evans, 1973). Histological studies referred to above have confirmed that this is so, and that the exception also extends to fibroblasts.

(2) Lines 257742 and 306462 were used at the third subculture generation. Cells from both these lines form colonies in soft agar, and line 257742 has been subcultured to the 34th subculture generation, at which stage it was inadvertently lost. Repeated subculture was not tried with line 306462. All these facts, taken together, suggest that at least some of the cells in these 2 lines were of malignant origin. BT20 is a cell line derived from a breast adenocarcinoma (Lasfargues and Ozzello, 1958) and it forms progressively growing tumours in athymic nude mice (Ozzello *et al.*, 1974).

None of the above statements conclusively shows that the target cells were derived from malignant breast epithelium. Unfortunately, the present state of scientific expertise in this area probably precludes a more definite answer. For what it is worth, the basic procedures employed have the sanction of fairly general usage, and the assumptions I have made about the nature of the target cells are not dissimilar to those made by most workers in this field.

The second question is whether the lymphocyte and serum effects seen were nonspecific effects or represented immune responses directed against antigenic moieties present in breast tumour cells. In a previous study (Jeejeebhoy, 1975), lymphoid cells from disease-free individuals and individuals with breast cancer were seeded on allogeneic breast tumour cells, melanoma cells and fibroblasts in a single experiment. Each of the lymphoid cell preparations was cytotoxic to breast tumour cells in the numbers used. Similar numbers of lymphoid cells were also occasionally cytotoxic to melanoma cells and fibroblasts. However, the pattern of the differences in the cytotoxic properties of lymphoid cells from the different individuals, *vis-à-vis* breast tumour cells, was not similarly expressed against melanoma cells and fibroblasts, albeit in different degree. In that study it was also found that humoral anti-breast tumour cell blocking and potentiating effects could be absorbed out with breast tumour cells but not with melanoma cells. All these facts, taken together, suggest that the effects seen when lymphoid cells and sera from disease-free individuals or individuals with breast cancer are seeded on breast tumour cells, are not nonspecific effects but rather represent the consequences of immune responses directed against antigens specific to breast tumour cells.

The third question is how sera which decreased lymphocyte anti-tumour cell cytotoxicity when applied to one tumour line, could potentiate or leave it unaltered when applied to another. The sera used could have contained immunologically specific substances directed against histocompatibility antigens (because of previous pregnancies, blood transfusions or recognized or unrecognized abortions) or tumour-associated antigens, and for these reasons any one or more of numerous factors could have been responsible for the differences noted: variations in the avidity of tumour cells (Hellstrom and Hellstrom, 1974) or lymphoid cells (Currie and Basham, 1972) for antibody or antigen–antibody complexes, quantitative differences in the amounts of free antibody, free antigen and/or free antigen–antibody complexes, *etc.* No consistent pattern of target-cell susceptibility to humoral effects was noted. It was therefore felt that further study would probably not yield any clear-cut answer, and was unlikely to be profitable.

In the context of this third question, it is noteworthy that there have been reports that lymphocyte effects against different tumour-cell lines having the same histological derivation, may not always be comparable (Mukherji *et al.*, 1975). The fact that they were in the present study may have been fortuitous, a consistent pattern being maintained from experiment to experiment because each of the tumour lines had been frozen in aliquots at a single point in time.

Finally, because of the problems involved in utilizing target cells derived from the autochthonous tumour, it is pertinent to ask whether those derived from allogeneic tumour-cell lines could serve as adequate substitutes. Most workers have assumed that they can (Herberman and Oldham, 1975) and the present results show that this is indeed acceptable, if ELAC levels are determined against not less than 3 lines, and the results not accepted unless ELAC levels against each of these 3 lines are comparable. These rather drastic caveats would have certainly eliminated all the discrepancies in the present study, but only at the cost of a large amount of labour and the failure to evaluate ELAC levels in a significant proportion of patients. Clearly then, the use of autochthonous tumour cells for determination of ELAC levels must remain the ideal.

A final note of pessimism is perhaps appropriate. The vast literature pertaining to *in vitro* studies of human anti-tumour immunity is characterized by results which are not regularly reproducible, and by correlations between *in vitro* data and clinical findings which are regularly demonstrable by some, and rarely by others (Herberman and Oldham, 1975). The present paper suggests one cause for these discrepancies.

I am indebted to my wife for her expert technical assistance, to Dr E. Lasfargues for supplying line BT20 cells and to the staff of the American Oncologic Hospital without whose help and collaboration none of this work would have been possible.

Part of this work was done while I was a recipient of an Eleanor Roosevelt Fellowship from the International Union against Cancer. This work was supported by U.S.P.H.S. grants CA-08856 and RR-05539 from the National Institutes of Health and by an appropriation from the Commonwealth of Pennsylvania.

### REFERENCES

Berkelhammer, J., Mastrangelo, M. J., Laucius, J. F., Bodurtha, A. J. & Prehn, R. T. (1975) Sequential *In vitro* Reactivity of Lymphocytes from Melamona Patients Receiving Immunotherapy Compared with the Reactivity of Lymphocytes from Healthy Donors. *Int. J. Cancer*, **16**, 571.

Currie, G. A. & Basham, C. (1972) Serum Mediated Inhibition of the Immunological Reactions of the Patient to his own Tumour: a Possible Role for Circulating Antigen. *Br. J. Cancer*, **26**, 427.

Evans, R. (1973) Preparation of Pure Culture of Macrophages. *J. natn. Cancer Inst.*, **50**, 271.

HELLSTROM, I., HELLSTROM, K. E., SJOGREN, H. O. & WARNER, G. A. (1971) Demonstration of Cell-mediated Immunity to Human Neoplasms of Various Histological Types. *Int. J. Cancer*, **7**, 1.

HELLSTROM, K. E. & HELLSTROM, I. (1974) Lymphocyte-mediated Cytotoxicity and Blocking Serum Activity to Tumor Antigens. *Adv. Immunol.*, **18**, 209.

HERBERMAN, R. B. & OLDHAM, R. K. (1975) Problems Associated with Study of Cell-mediated Immunity to Human Tumors by Microcytotoxicity Assays. *J. natn. Cancer Inst.*, **55**, 749.

JEEJEEBHOY, H. F. (1974) Stimulation of Tumor Growth by the Immune Response. *Int. J. Cancer*, **13**, 665.

JEEJEEBHOY, H. F. (1975) Immunological Studies of Women with Primary Breast Carcinoma. *Int. J. Cancer*, **15**, 867.

JEEJEEBHOY, H. F. & LAWLER, E. M. (1977) The *In vitro* Manifestations of Cellular and Humoral Allograft and Anti-tumor Immunity are not Appreciably Altered by Previous Storage of Target and Effector Cells in Liquid Nitrogen. *J. Reticuloendothel. Soc.*, (in press).

LASFARGUES, E. Y. & OZZELLO, L. (1958) Cultivation of Human Breast Carcinomas. *J. natn. Cancer Inst.*, **21**, 1131.

MAVLIGIT, G. M., GUTTERMAN, J. U. & HERSH, E. M. (1973) Separation of Viable from Non-viable Tumor Cells using Ficoll-Hypaque Density Solutions. *Immunol. Commun.*, **2**, 463.

MUKHERJI, B., VASSOS, D., FLOWERS, A., BINDER, S. C. & NATHANSON, L. (1975) Variables and Specificity of *In vitro* Lymphocyte-mediated Cytotoxicity in Human Melanoma. *Cancer Res.*, **35**, 3721.

OZZELLO, L., SORDAT, B., MERENDA, C., CARREL, S., HURLIMANN, J. & MACH, J. P. (1974) Transplantation of a Human Mammary Carcinoma Cell Line (BT 20) into Nude Mice. *J. natn. Cancer Inst.*, **52**, 1669.

SKURZAK, H. M., KLEIN, E., YOSHIDA, T. O. & LAMON, E. W. (1972) Synergistic and Antagonistic Effect of Different Antibody Concentrations on *In vitro* Lymphocyte Cytotoxicity in the Moloney Sarcoma Virus System. *J. exp. Med.*, **135**, 997.

TAKASUGI, M. & KLEIN, E. (1970) A Microassay for Cell-mediated Immunity. *Transplantation*, **9**, 219.

TAKASUGI, M., MICKEY, M. R. & TERASAKI, P. I. (1973) Reactivity of Lymphocytes from Normal Persons on Cultured Tumor Cells. *Cancer Res.*, **33**, 2898.